# Three-Dimensional Measurements in Assessing the Results of Inferior Turbinate Surgery

**DOI:** 10.1177/00034894211028516

**Published:** 2021-07-01

**Authors:** Olli Valtonen, Jaakko Ormiskangas, Teemu Harju, Markus Rautiainen, Ilkka Kivekäs

**Affiliations:** 1 Department of Otorhinolaryngology – Head and Neck Surgery, Tampere University Hospital, Tampere, Finland; 2 Faculty of Medicine and Health Technology, Tampere University, Tampere, Finland; 3 Faculty of Engineering and Natural Sciences, Automation Technology and Mechanical Engineering Unit, Tampere University, Tampere, Finland

**Keywords:** 3D, volumetry, nasal cavity, turbinate, RFTA

## Abstract

**Objectives:**

Acoustic rhinometry is widely used in evaluating patients with nasal congestion, but it only has a partial correlation with patient symptoms. The use and focus of cone beam computed tomography (CBCT) scans are mainly on the paranasal sinuses and less on the nasal cavities. Therefore, information acquired from CBCT scans is not used to its full extent. In our present study, we have studied patients with enlarged inferior turbinates. Our aim was to investigate and compare the use of 3D volumetric measurements and cross-sectional area measurements taken from CBCT scans to results obtained from acoustic rhinometry.

**Material and methods:**

In total, 25 patients with enlarged inferior turbinates were studied. CBCT scans were obtained preoperatively and at twelve months postoperatively. 3D volumetric and cross-sectional area measurements were compared to results from acoustic rhinometry, the visual analogue scale (VAS) and Glasgow Health Status Inventory (GHSI) questionnaires.

**Results:**

A statistically significant change in 3D volume and cross-sectional area was measured in the anterior part of the inferior turbinate and surrounding air space after inferior turbinate surgery. VAS and GHSI results had mild correlations with the 3D volume and cross-sectional area measurements of the anterior part of the inferior turbinate. Acoustic rhinometry correlated with the air space 3D volume measurements in the anterior part.

**Conclusions:**

Fully utilized CBCT scans provide more comprehensive and accurate information. Furthermore, 3D analysis of the inferior turbinates provides valuable information and more precise measurements compared to acoustic rhinometry.

## Introduction

Today, the 2 most common objective methods for assessing nasal congestion are acoustic rhinometry and rhinomanometry.^1,2^ Both methods have proven to be fast and mostly reliable in measuring dimensions and breathing resistance in the nose. The use of these methods has, however, been problematic, especially when the patient’s subjective sensations of nasal blockage or patency are taken into account. Moreover, the Visual Analogue Scale (VAS) and other symptom questionnaires have failed to show a consensus of correlation with acoustic rhinometry or rhinomanometry.^3^

In previous studies, the assessment of the volume of the nasal cavities has been mostly done using information gained from acoustic rhinometry. However, acoustic rhinometry, especially in the posterior regions, is known to overestimate the dimensions of the nasal cavity.^4-7^ Indeed, in a study by Cankurtaran et al, acoustic rhinometry was found to have overestimated the volume of the nasal cavity airway by more than 20%. To date, however, the actual 3D volumetric measurements from pre- and postoperative CT or magnetic resonance imaging (MRI) scans and any possible benefits compared to acoustic rhinometry in patients with operated nasal cavities have not been extensively studied.

Inferior turbinate surgery with different methods is one of the main surgical procedures for treating nasal congestion. Usually, acoustic rhinometry and different subjective assessment methods are chosen to evaluate the effects of these operations on the circumstances in the nasal cavity. In previous studies, the postoperative follow-up times have been fairly short, and the most commonly used time span between pre- and postoperative data gathering has varied from a few weeks to a few months.^8^ The long-term effects of these operations on volumetric dimensions, acoustic rhinometry results, patient symptoms and their correlations from 1 or more years of follow-up have not as yet been studied sufficiently.

In the present study, the aim was to study and compare the use of 3D volumetric measurements accompanied with cross-sectional area measurements to those results obtained from acoustic rhinometry. Moreover, the potential of 3D volumetric and cross-sectional area measurements in reflecting the patients’ subjective sensations was also studied using VAS and quality of life (QOL) scores.

## Materials and Methods

In the present study, 26 patients with chronic nasal obstruction were included. These patients had enlarged inferior turbinates and underwent radiofrequency thermal ablation (RFTA) treatment (Sutter RF generator BM-780 II, Freiburg, Germany) to the inferior turbinates on both sides. The patients were scanned preoperatively and at twelve months postoperatively with cone beam computed tomography (CBCT) (Planmeca Max, Planmeca, Helsinki, Finland).

CBCT data were saved to a file in Digital Imaging and Communications in Medicine (DICOM) format and downloaded to OnDemand3D™ software (version 1.0, CyberMed, Inc., Yuseong-gu, Daejeon, South Korea). OnDemand3D™ software was used to perform the 3D volumetric and cross-sectional area measurements.

**Figure 1.**
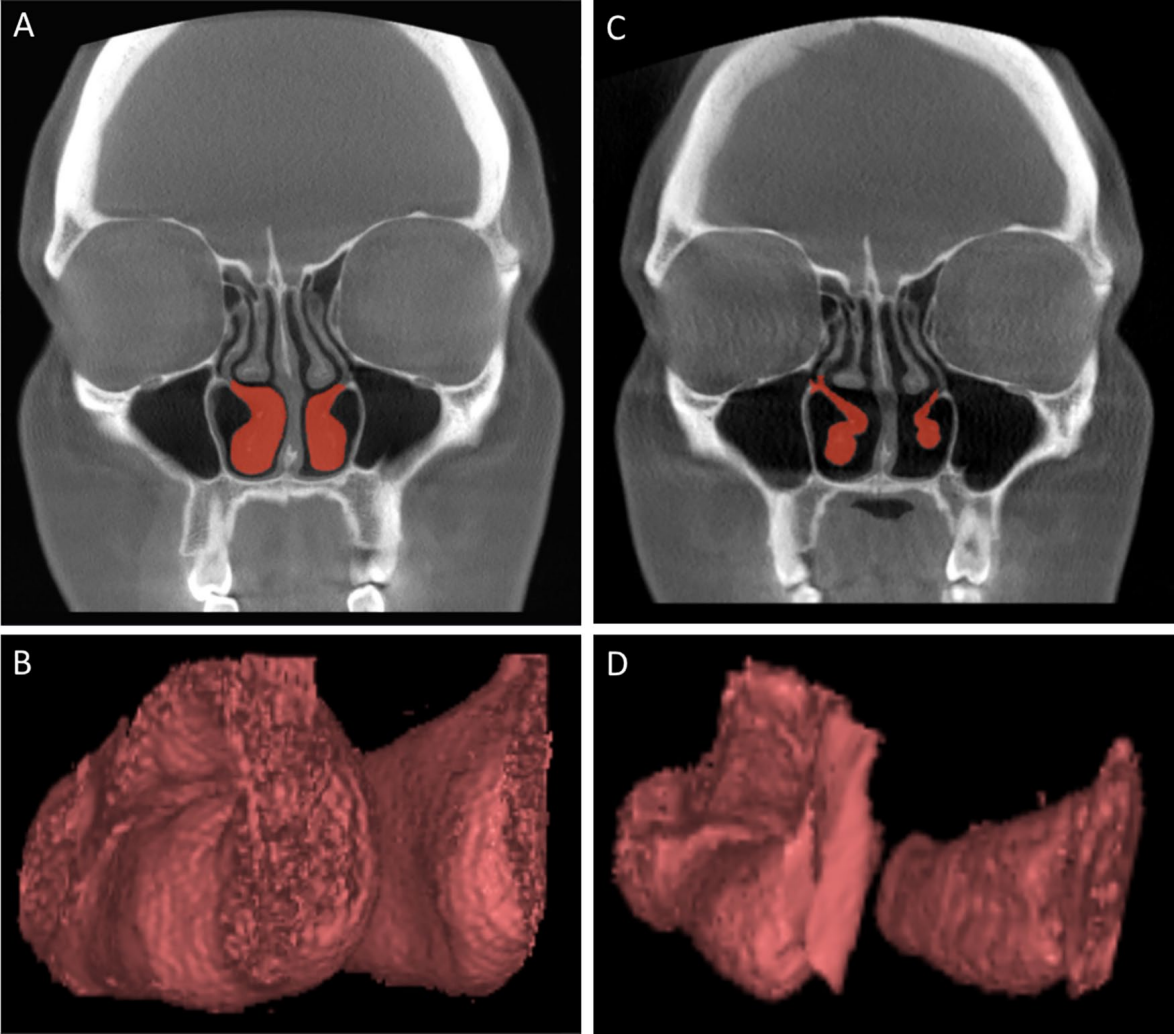
An example of the preoperative patient CBCT scans (A) with marked inferior turbinates (red) used as a basis for the production of the inferior turbinate 3D models (B) which were used in the 3D volumetric measurements. Corresponding examples of the postoperative patient CBCT scans (C) and the produced inferior turbinate 3D models (D) are also presented.

**Figure 2.**
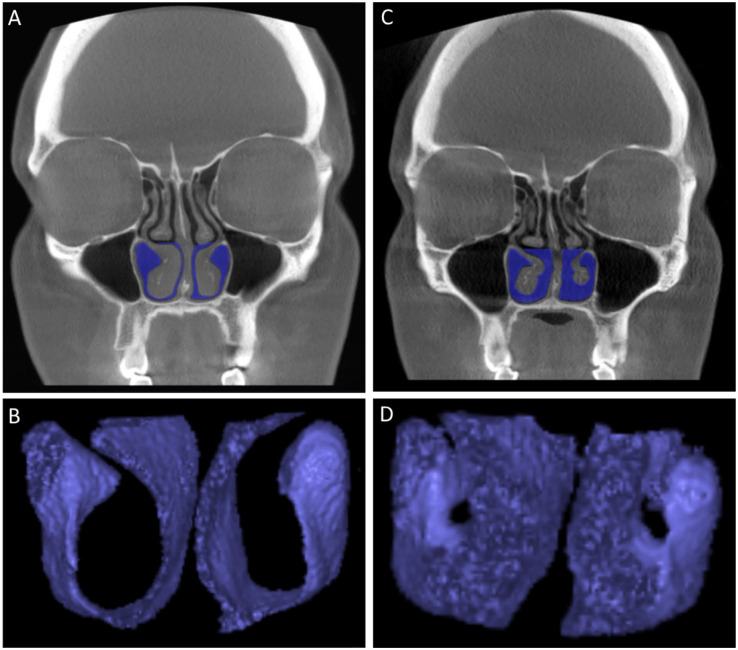
An example of the preoperative patient CBCT scans (A) with marked air spaces (blue) surrounding the operated anterior parts of the inferior turbinates used as a basis for the production of the air space 3D models (B) which were used in the 3D volumetric measurements. Corresponding examples of the postoperative patient CBCT scans (C) and the produced air space 3D models (D) are also presented.

On both sides, the volume of the whole inferior turbinate was 3D modeled and measured from the pre- and postoperative CBCT scans (Figure 1). The same measurements were performed on the operated anterior part of the inferior turbinate and the air space surrounding the turbinate (Figure 2). The anterior part consisted of an area from 5 to 20 mm posterior from the anterior peak of the inferior turbinate. Hounsfield Unit (HU) values from −429 to 400 were used to measure the inferior turbinate. These values were obtained using the measuring software’s own scaling function. HU values from −1000 to −430 were used for the measurement of the pneumatized area according to previous studies.^9-11^ Some 3D modeling artefacts, included to the 3D measurements by the software, were manually excluded from the structures of interest.

We also used CBCT scans to measure the coronal cross-sectional areas from both the inferior turbinates and the air spaces surrounding the inferior turbinates. The measurement of the cross-sectional areas started 5 mm and ended 50 mm posterior from the anterior peak of the inferior turbinate. The distance between each measuring point was 5 mm. For these measurements, we used the Smart Pen function of OnDemand3D™ with some manual corrections.

For both the volumetric and cross-sectional area measurements, the uppermost limit of the region of interest (ROI) was set at the lowest level of the middle turbinates. The nasal cavity floor was used as the lowest level of the ROI. Using the OnDemand3D™ software, the volumetric and cross-sectional area measurements of the pre- and postoperative CBCT scans took approximately 7 to 10 hours per patient.

The patients were asked to fill in the VAS questionnaire preoperatively and at 12 postoperatively in order to assess the severity of nasal obstruction. To assess the effects of a health problem on quality of life, the patients were asked to fill in the Glasgow Health Status Inventory (GHSI) questionnaire. Acoustic rhinometry (Acoustic rhinometer A1, GM instruments Ltd, Kilwinning, UK) was also performed pre- and postoperatively. An encompassing analysis of the acoustic rhinometry and subjective questionnaire results has been presented in a previous study.^12^ The acoustic rhinometry tests without adrenaline and the results from both MCA2 and V2-5 were used in this study.

Before the final data analysis, 1 patient was excluded from the study due to extensive artefacts in the CBCT scans caused by dental fillings which prevented 3D measurements. Thus, 25 patients were included in the present study’s data analysis. In addition, with regard to only the correlation analysis, 1 patient had to be excluded due to missing acoustic rhinometry measurements. This resulted in a total of 24 patients being included in this analysis.

All the data were analyzed using SPSS (version 26, IBM, Armonk, NY, USA) software. The Wilcoxon signed-rank test was used to analyze the statistical significance of the measurement results. The Spearman correlation test was used for correlation analysis.

Institutional Review Board approval for the study (R13144) was obtained from the Ethics Committee of Tampere University Hospital, Tampere, Finland.

## Results

The preoperative median total volume from the combined left and right inferior turbinates was 11.7 cm^3^. The median total volume postoperatively was 9.3 cm^3^. In the anterior 5 to 20 mm of the inferior turbinate, the preoperative inferior turbinate total volume of 4.2 cm^3^ decreased postoperatively to 3.0 cm^3^. Corresponding volumetric results for the pneumatized area in the anterior part increased from 2.3 to 3.4 cm^3^. The changes between preoperative and postoperative volumes were statistically significant in all the measured areas (Table 1).

**Table 1.** Median volumetric pre- and postoperative results. Turbinate volume represents the inferior turbinate as a whole and the anterior part measurements are presented in the 5 to 20 mm results (n = 25). The results are shown from both sides separately and the total results represent the combined results from both the left and right sides. The lower quartile (Q_25_) and upper quartile (Q_75_) are shown with the median. The statistical significance of the change between pre- and postoperative results, analysed with Wilcoxon signed-rank test, is also disclosed.

	Median volume (cm^3^)						
	Left (pre)	Right (pre)	Total (pre)	Left (post)	Right (post)	Total (post)	Total (change)
Turbinate	4.9	5.6	11.7	4.8	4.5	9.3	−1.7
	Q_25_ = 3.8	Q_25_ = 4.6	Q_25_ = 9.0	Q_25_ = 3.9	Q_25_ = 3.5	Q_25_ = 8.0	Q_25_ = −3.3
	Q_75_ = 6.8	Q_75_ = 7.2	Q_75_ = 13.1	Q_75_ = 6.3	Q_75_ = 5.7	Q_75_ = 11.2	Q_75_ = 0.0
							*P* < .01
Turbinate (5-20 mm)	2.1	2.2	4.2	1.6	1.4	3.0	−1.2
	Q_25_ = 1.4	Q_25_ = 1.6	Q_25_ = 3.3	Q_25_ = 1.3	Q_25_ = 1.3	Q_25_ = 2.7	Q_25_ = −2.0
	Q_75_ = 2.6	Q_75_ = 2.6	Q_75_ = 4.9	Q_75_ = 2.0	Q_75_ = 1.6	Q_75_ = 3.3	Q_75_ = −0.4
							*P* < .001
Air (5-20 mm)	1.4	1.2	2.3	1.6	1.9	3.4	0.8
	Q_25_ = 0.9	Q_25_ = 0.7	Q_25_ = 2.0	Q_25_ = 1.2	Q_25_ = 1.5	Q_25_ = 2.7	Q_25_ = 0.2
	Q_75_ = 1.8	Q_75_ = 1.6	Q_75_ = 3.4	Q_75_ = 2.2	Q_75_ = 2.3	Q_75_ = 4.4	Q_75_ = 1.9
							*P* < .001

**Figure 3.**
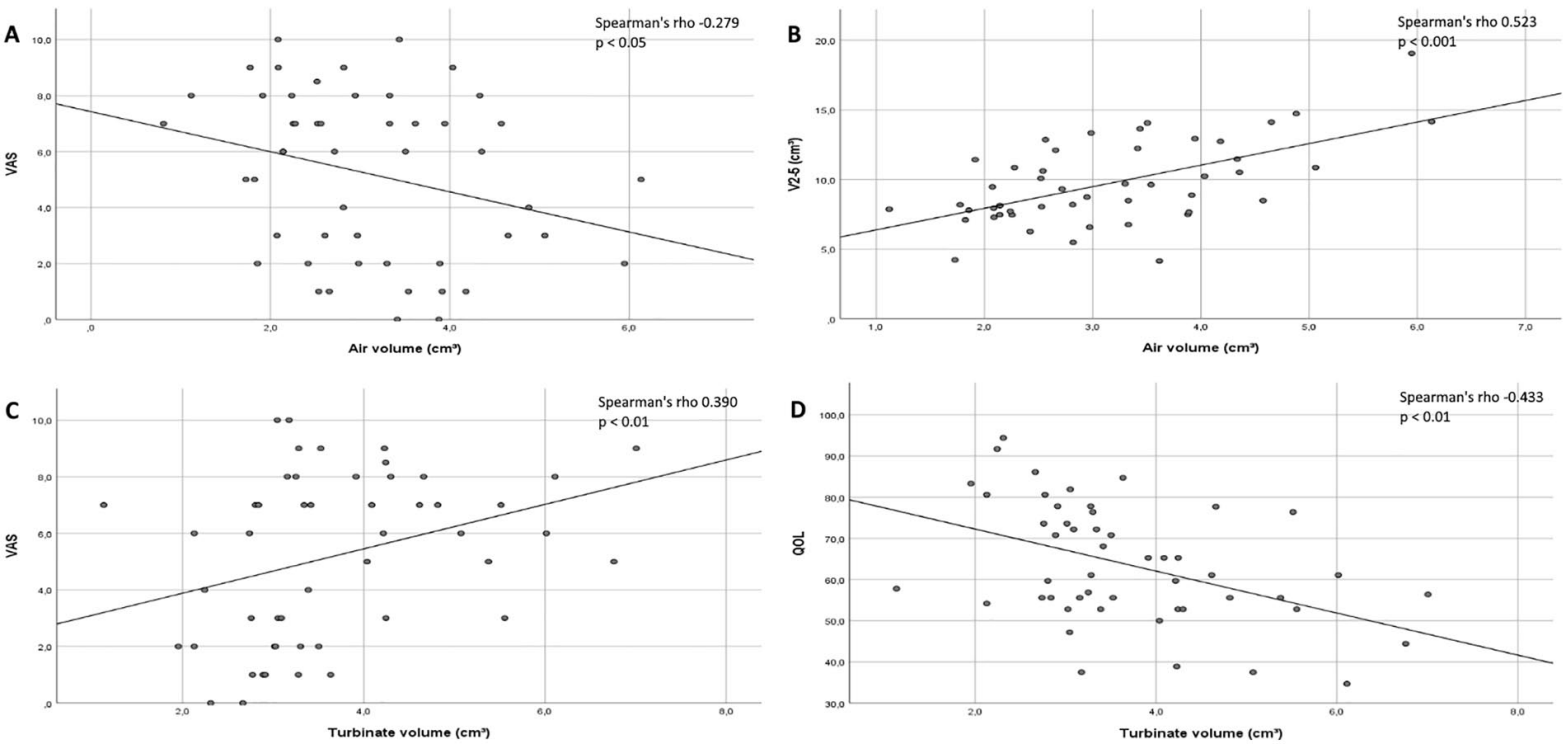
Correlation graphs for the 3D volumetric measurements of the individual anterior parts of the inferior turbinates (n = 48). The air volume (cm^3^) correlations between VAS score and V2-5 (cm^3^) are presented in graphs (A) and (B). The turbinate volume (cm^3^) correlations between VAS and QOL scores are presented in graphs (C) and (D). Spearman’s rho number and R^2^ correlation line are included in the graphs.

When the preoperative and postoperative measurements were analyzed together, comprising 48 cases in total, the volumetric measurements of the pneumatized nasal cavity in the anterior part of the measured region correlated with V2-5 results from acoustic rhinometry (0.523, *P* < .001) and with VAS scores (−0.279, *P* < .05) (Figure 3). Turbinate volume correlated with VAS (0.390, *P* < .001) and QOL (−0.433, *P* < .001) scores in the anterior part of its volume. However, the measurements of the whole length of the inferior turbinate or the changes in it did not correlate with the other parameters. Moreover, the volume changes in the anterior part of the inferior turbinate or the air space surrounding it did not correlate with changes in the other parameters.

**Figure 4.**
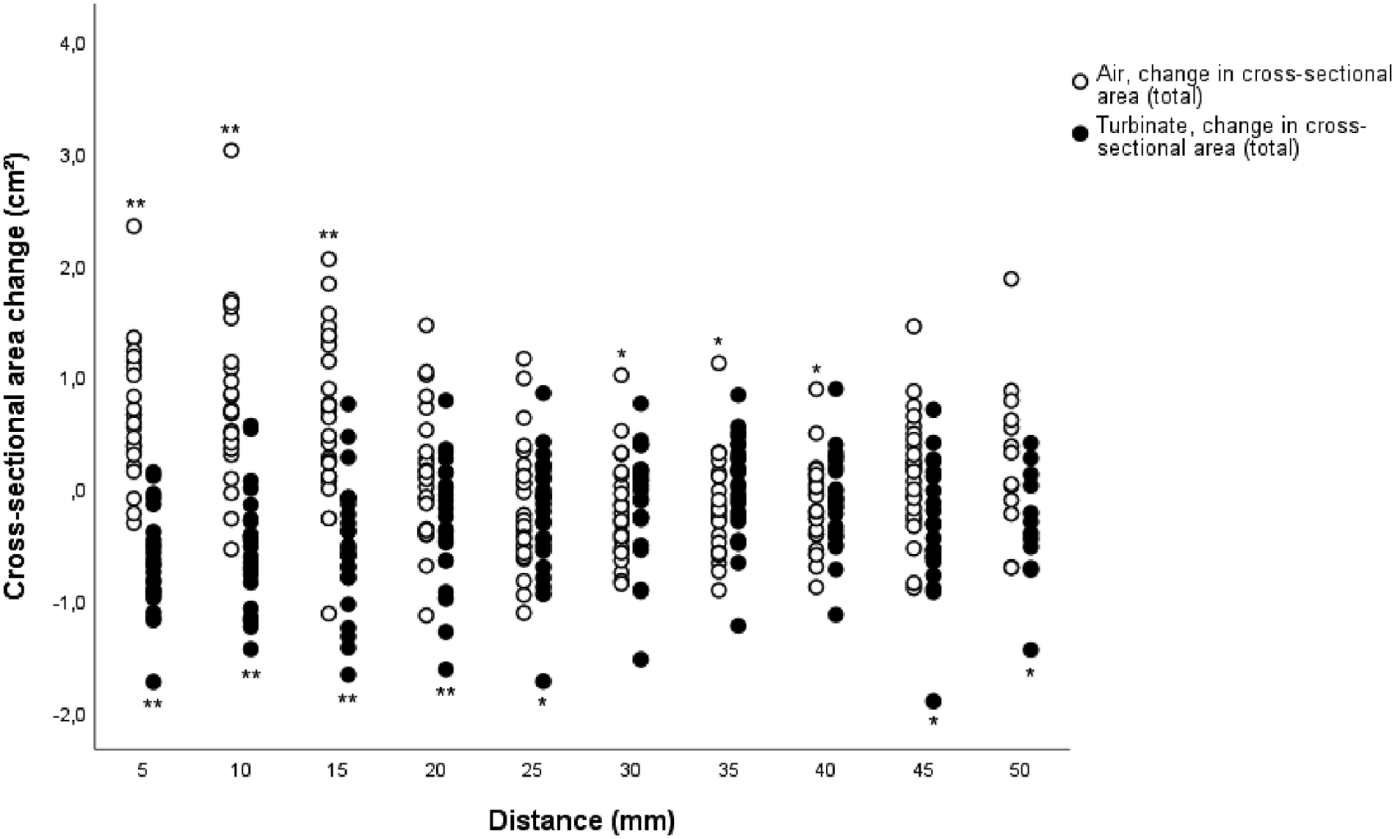
Cross-sectional area changes in both inferior turbinate and surrounding air in every measuring point (n = 25). The total results represent the combined left and right nasal cavity results. The distance represents the measuring point distance from the anterior peak of the inferior turbinate. Statistically significant inferior turbinate or air space changes per every measuring point, analysed with Wilcoxon signed-rank test, are marked with an asterisk. *P < .05. **P < .01.

The main cross-sectional area changes from pre- to postoperative values were found in the region of the operated anterior part of the inferior turbinate and the pneumatized area surrounding it (Figure 4). These changes were also statistically significant. Some smaller cross-sectional area changes with a statistical significance were also found in the posterior regions of the inferior turbinate. When the pre- and postoperative measurements were analyzed together, the cross-sectional area measurements correlated with VAS and QOL scores in the most anterior measurement points but did not correlate with the other parameters (Table 2). The cross-sectional area changes did not correlate with changes in the other parameters.

**Table 2.** Cross-sectional area correlation with VAS and QOL scores in the area of the anterior part of the inferior turbinate for both pre- and postoperative results (n = 48). The correlation coefficient with VAS and QOL is shown for every cross-sectional area measuring point from the anterior part (5-20 mm) of the inferior turbinate. The statistically significant results, analysed with Spearman’s rho test, are presented with an asterisk.

Cross-sectional area correlation with VAS and QOL								
	Air				Turbinate			
	5 mm	10 mm	15 mm	20 mm	5 mm	10 mm	15 mm	20 mm
VAS	−0.408**	−0.411**	−0.261	0.006	0.539**	0.439**	0.379**	0.242
QOL	0.280*	0.323*	0.242	−0.031	−0.400**	−0.377**	−0.411**	−0.251

* *P* < .05. **P < .01.

## Discussion

At present, CBCT scans are widely used as part of the examination of patients with symptoms of nasal congestion. The information gained from these scans, however, is not used to its full extent. In our present study, we demonstrate the use of 3D volumetric measurements combined with cross-sectional area measurements to assess the results of operative treatment in the inferior turbinates from CBCT scans. These methods proved to be accurate in measuring the inferior turbinate and the surrounding air space and in assessing the changes in them. Moreover, the measurements produced both objective and descriptive results on the effects of the RFTA treatment.

In the whole length of the inferior turbinate, the change from pre- to postoperative volume was −15%. A corresponding volume change for the anterior part of the inferior turbinate was −28% and +41% for the surrounding air space. These findings reflect well the given treatment’s effect on the operated turbinates, where the anterior part of the inferior turbinates is treated.

Some changes in the inferior turbinate and the surrounding air cross-sectional area measurements were also found in the middle and posterior measuring points (Figure 4). In the middle regions, for example, both the cross-sectional area of the turbinates and the air had decreased. In addition, even though the most posterior regions of the inferior turbinate were not operated, the effect of the operation seemed to be parallel, though statistically significant at a mild level, to the anterior part of the inferior turbinate. It is possible therefore that these changes could be due to a neural or vascular process in the inferior turbinate and possibly even in the whole nasal cavity. These findings suggest that other compensatory changes might occur in the other parts of the nasal cavity after inferior turbinate surgery that could subsequently have an effect on the patients’ sensation of nasal obstruction or patency. However, our study focused only on the inferior turbinates and their surroundings, and therefore further studies are required to assess this possible phenomenon.

We found correlations between the volume of the pneumatized area around the anterior part of the inferior turbinates and both the V2-5 results from acoustic rhinometry and VAS scores, although these correlations were mild (Figure 3). Both subjective assessment methods had a mild correlation with the anterior turbinate volume measurements. These findings are in line with the observations of previous studies, where the subjective measurement methods did not produce strong correlating results with objective clinical assessment methods.^3,13-16^ In these previous studies, VAS scale results correlated with results that varied from no correlation to fairly good correlation when compared to acoustic rhinometry or rhinomanometry.

We are aware of only a few studies in which the volumetric measurements of the nasal cavity structures taken from CT or MRI scans have been used alongside results from acoustic rhinometry to assess operated nasal cavity volumes. Kilavuz et al^17^ studied electrocautery and radiofrequency tissue reduction in the treatment of inferior turbinate hypertrophy and found the effects of both operative methods on inferior turbinate volumes to be close to each other when measured from MRI scans. However, the correlation of the MRI scan measurements with the results from acoustic rhinometry were not compared. A study by Numminen et al^18^ compared nasal cavity volume measurements from high-resolution computed tomography (HRCT) scans with acoustic rhinometry. They came to the conclusion that acoustic rhinometry produces good results in the anterior and middle parts of the nasal cavity but weaker results in the posterior parts. However, the study used patients undergoing uncinectomy without any turbinate operations. One study measuring actual nasal cavity 3D volumes from CT scans concentrated mainly on evaluating the results of septorhinoplasty without assessing the correspondence of the volumetric measurements with other used methods, such as VAS, acoustic rhinometry or rhinomanometry.^19^ The study showed that septorhinoplasty caused a significant increase in nasal cavity volume, which was demonstrated with 3D measurements.

The results of inferior turbinate surgery have previously been studied by measuring the whole inferior turbinate volume and concentrating on the acquired change in it.^17,20^ These studies have used either CT or MRI scans to measure and compare inferior turbinate volumes pre- and postoperatively. In a study by Bozan et al, inferior turbinate volumes were calculated from linear measurements. In the study by Kilavuz et al, however, the technique used for inferior turbinate volume measurements was not clearly described. The only method used to assess the parameters of the nasal cavity air space was acoustic rhinometry. Unfortunately, these studies did not assess how well these 2 different methods correlated with each other or how accurate they were. Thus, the results are not fully comparable with our 3D measurements.

Although measuring nasal cavity air space dimensions with acoustic rhinometry is relatively reliable and fast to perform, the method has its limitations and uncertainties. In previous studies by Cankurtaran et al,^4^ Hilberg et al^5^ and Terheyden et al,^6^ the acoustic rhinometry results considerably overestimated the cross-sectional areas in, and especially after the mid parts of the nasal cavity when compared to the actual CT measurements. This has been interpreted to be caused by the sound loss through the ostia to the paranasal sinuses or by the interaction of the nasal cavity and the paranasal sinuses. Therefore, the possible changes in the middle and posterior parts of the nasal cavity cannot be reliably assessed with acoustic rhinometry, which means that possible significant changes in the nasal cavities remain unobserved. Our finding that V2-5 results had a mild correlation with only the volumetric measurements of the anterior region support this finding (Figure 3).

The limitation of the methods we used in our study is that they are still time consuming. However, combined information from the 3D volumetric method and cross-sectional area measurements provide an objective method for assessing the results of inferior turbinate surgery and other operations of the nasal cavity. As previously mentioned, the assessment results are more precise than those provided by acoustic rhinometry, especially in the mid and posterior parts of the nasal cavity.

Today, the 3D volumetric method combined with cross-sectional area measurements are only applicable for study purposes, and more studies are needed to adjust and speed up the process. The comprehensive assessment of nasal cavity anatomy, changes to it and the effect they have on nasal cavity airflows and patient symptoms will most likely require studies that are carried out using 3D modeling software that can 3D model the airflow conditions and the effect on the mucous membrane.

## Conclusions

A significant 3D volume and cross-sectional area decrease was measured in the anterior part of the inferior turbinate after RFTA treatment, whereas the surrounding air increased significantly. The treatment led to some possible compensatory changes, especially in the middle and posterior parts of the inferior turbinates. Overall, 3D volumetric and cross-sectional area measurements had mild to moderate correlation with other parameters. In evaluating the effects of inferior turbinate surgery, 3D volumetric and cross-sectional area measurements proved to be accurate. CBCT scans can be used more comprehensively as a diagnostics tool and for further analytics.
